# Effects of *Coptis* extract combined with chemotherapeutic agents on ROS production, multidrug resistance, and cell growth in A549 human lung cancer cells

**DOI:** 10.1186/1749-8546-7-11

**Published:** 2012-04-30

**Authors:** Chengwei He, Rong Rong, Jing Liu, Jianbo Wan, Keyuan Zhou, Jing X Kang

**Affiliations:** 1 Department of Medicine, Massachusetts General Hospital and Harvard Medical School, Charlestown, Boston, MA, 02129, USA; 2 State Key Laboratory of Quality Research in Chinese Medicine, University of Macau, Macao, SAR, China; 3 Institute of Chinese Medical Sciences, University of Macau, Macao, SAR, China; 4 Department of Nephrology, The First Affiliated Hospital of Sun Yat-sen University, Guangzhou, Guangdong, China; 5 Biochemistry and Molecular Biology Institute, Guangdong Medical College, Zhanjiang, Guangdong, China

## Abstract

**Background:**

Non–small cell lung cancer is associated with high expression of multidrug resistance (MDR) proteins and low production of reactive oxygen species (ROS). *Coptis* extract (COP), a Chinese medicinal herb, and its major constituent, berberine (BER), have anticancer properties. This study aims to investigate the effects of COP and BER combined with chemotherapeutic agents, including fluorouracil (5-FU), camptothecin (CPT), and paclitaxel (TAX), on cell proliferation, ROS production, and MDR in A549 human non-small cell lung cancer cells.

**Methods:**

A549 cells were treated with different doses of COP and BER, combined with 5-FU, CPT, and TAX. Cell viability was measured by an XTT (2,3-bis-(2-methoxy-4- nitro-5-sulfophenyl)-2 H-tetrazolium-5-carboxanilide) assay. Intracellular ROS levels were determined by measuring the oxidative conversion of cell permeable 2′,7′-dichlorofluorescein diacetate to fluorescent dichlorofluorescein. MDR of A549 cells was assessed by rhodamine 123 retention assay.

**Results:**

Both COP and BER significantly inhibited A549 cell growth in a dose-dependent manner. Combinations of COP or BER with chemotherapeutic agents (5-FU, CPT, and TAX) exhibited a stronger inhibitory effect on A549 cell growth. In addition, COP and BER increased ROS production and reduced MDR in A549 cells.

**Conclusion:**

As potential adjuvants to chemotherapy for non–small cell lung cancer, COP and BER increase ROS production, reduce MDR, and enhance the inhibitory effects of chemotherapeutic agents on A549 cell growth.

## Background

The herb *Coptis* (COP) is used to treat “damp heat” syndrome in Chinese medicine [1]. Its major constituent is berberine (BER), an isoquinoline alkaloid [2]. The anticancer effects of COP and BER on both hematological and nonhematological cancers have been well documented [3]. Since 2000, experimental studies have confirmed the cytotoxicity of BER in various cancer cell lines, including YES (esophageal carcinoma) [4], HK1 (nasopharyngeal carcinoma) [5], HeLa (cervical carcinoma) [6], HepG2 (hepatocellular carcinoma) [7]. Our previous studies [9,10] have also shown that COP inhibits the growth of breast cancer cells.

Non–small cell lung cancer (NSCLC) accounts for approximately 85% of lung cancers, and only responds to 15%–25% single agents and 25%–40% combined chemotherapy [11]. NSCLC is typically resistant to apoptosis induced by standard chemotherapy, which causes excessive levels of reactive oxygen species (ROS), leading to impaired intracellular ionic homeostasis by damaging cellular macromolecules and inducing apoptosis [12]. Mitochondrial ROS production is crucial to NSCLC apoptosis induced anticancer agents [13]. In addition to ROS, multidrug-resistance (MDR) proteins are intrinsically expressed and functionally active in NSCLC cells [14]. Several adjuvants to chemotherapy for NSCLC are being tested, with promising results, including the antagonists EGFR and COX-2, [15-17].

This study aims to investigate the effects of COP and BER on ROS production and MDR, and the effects of combinations of COP or BER with chemotherapeutic agents, including fluorouracil (5-FU), camptothecin (CPT), and paclitaxel (TAX) on A549 human cancer cells, which are derived from NSCLC [18].

## Methods

### Materials

A powder form of COP extract was made from *Coptis japonica* (Mayway Corporation, Oakland, CA, USA) by boiling the plant in water and spray drying. A solution of COP was prepared as previously described [10]. BER, 5-FU, CPT, and TAX were purchased from Sigma-Aldrich (USA). BER, 5-FU, and CPT were dissolved in dimethylsulfoxide (DMSO) (Sigma-Aldrich, USA), and TAX was dissolved in 100% ethanol (Fisher Scientific, USA). The final concentrations of DMSO and ethanol in the medium were less than 0.1%.

### Cell culture

The A549 cell line (ATCC CCL-185) was purchased from ATCC (Manassas, VA, USA). The cells were cultured in Dulbecco's Modified Eagle Medium (DMEM) (Invitrogen, Carlsbad, CA, USA) supplemented with 100 U/mL penicillin, 100 g/mL streptomycin, and 10% inactivated fetal calf serum (FBS, HyClone, South Logan, UT, USA). The cells were incubated at 37°C under a humidified atmosphere of 95% air and 5% CO_2_. Cells were subcultured twice weekly.

### Cell viability assay

A549 cells were seeded in 96-well plastic plates (3 × 10^3^ cells/well) and incubated at 37°C in complete medium for 16 h before the drug treatment for 72 h. Cell viability was assessed by the XTT (2,3-bis-(2-methoxy-4- nitro-5-sulfophenyl)-2 H-tetrazolium-5-carboxanilide) assay (Invitrogen, Carlsbad, CA, USA) . The spectrophotometric absorbance of the samples was measured using a microplate reader (VICTOR^3^ V™ 1420 Multilabel Counter, PerkinElmer, Waltham, MA, USA) at 490 nm with a reference wavelength of 690 nm. Each measurement was performed in triplicate and the data reported were mean values of at least 3 experiments. The inhibitory effect was calculated according to the following equation:

(1) $$\text{Inhibition}\left(\%\right)=\left[1-\left(A490\text{ of treated wells}/\text{A}490\text{ of control wells}\right)\right]\times 100.$$

### Measurement of reactive oxygen species

Intracellular ROS levels were determined by measuring the oxidative conversion of cell permeable 2′, 7′ dichlorofluorescein diacetate (DCFH-DA; Sigma-Aldrich, USA) to fluorescent dichlorofluorescein (DCF) [13]. Cells were plated in 24-well culture plates (2 × 10^5^ cells/well) and incubated with drugs for 24 h. The cells were then washed with d-Hank’s solution (Invitrogen, USA) and incubated with 10 μM DCFH-DA in phenol-red-free MEM medium (Invitrogen, USA) at 37°C for 15 min. DCF fluorescence distribution was measured in a microplate reader (PerkinElmer, Waltham, MA, USA) at an excitation wavelength of 488 nm and emission wavelength of 535 nm. The fluorescence intensity was normalized according to the number of cells.

### Rhodamine 123 retention assay

Rhodamine 123 retention was measured to evaluate the multidrug resistance (MDR) of cancer cells [19,20]. Briefly, 24-well plates with 2 × 10^5^ cells/well were treated with low or high doses of COP (low dose: 1.6 μg/mL, high dose: 6.4 μg/mL) and BER (low dose: 0.5 μg/mL, high dose: 4 μg/mL) for 24 h. The cells were then incubated in phenol-red-free MEM containing 10 μg/mL rhodamine 123 (Sigma-Aldrich, USA) and 2% FBS for 20 min at 37°C. After incubation, the cell monolayers were washed 3 times with ice-cold phosphate buffered saline (PBS), trypsinized, and suspended in MEM medium containing 2% FBS. The cell suspension was transferred to a 96-well plate and measured in a microplate reader (PerkinElmer,Waltham, MA, USA) at an excitation wavelength of 485 nm and emission wavelength of 535 nm. The fluorescence intensity was normalized by the number of cells. All experiments were performed in quadruplicate.

### Statistical analysis

The data were expressed as means ± standard deviations (SD). A one-way analysis of variance (ANOVA) was performed to test the difference among groups of controls, individual agents, and combinations of agents using Graph Pad Prism 4 software (San Diego, CA, USA). Newman-Keuls test were used for multiple comparisons. The software did not provide exact *P* values for ANOVA; thus, no exact P values are reported. The results with *P* < 0.05 were considered statistically significant. Each experiment was repeated at least 3 times.

## Results

### A549 cell growth

**Figure 1:**
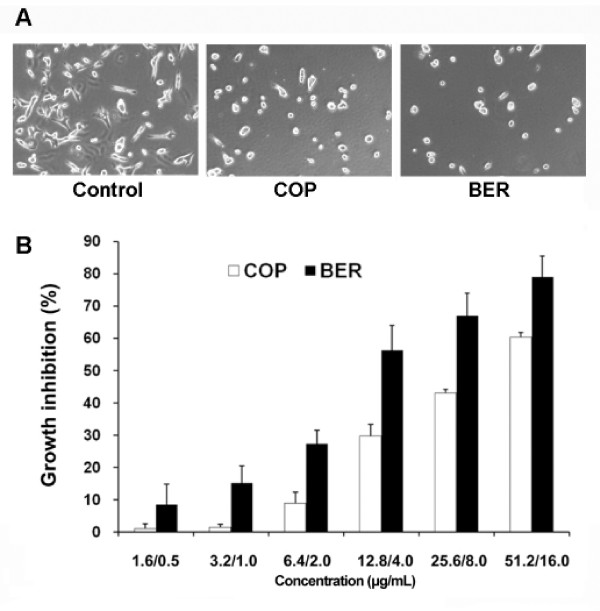
**Effects of COP and BER on the growth of A549 cells. A**. Morphological changes of A549 cells treated with 6.4 μg/mL COP or 4.0 μg/mL BER for 72 h under inverted phase contrast microscope (200 ×). **B**. Treatment with COP and BER for 72 h inhibited A549 cell growth (*P* < 0.01) [Pearson Correlation Test, *P* = 0.0032 for COP; *P* = 0.0178 for BER] in a dose-dependent manner. Values are means ± SD of 4 independent assays.

The A549 cell line was treated with different doses of COP and BER and tested for cell viability with an XTT assay to examine the effects of COP and BER on cancer cell growth. As shown in Figure 1, treatment with COP at 6.4 to 51.2 μg/mL or BER at 2.0 to 16.0 μg/mL for 72 h significantly inhibited A549 cell growth. The maximum inhibition rates were 60% and 64% for COP and BER, respectively. A Pearson Correlation Test by Prism 4 was used to determine the correlation between the doses of COP or BER and the inhibitory effects on A549 cell growth. The results indicated that the growth inhibition was in a dose-dependent manner [*P* = 0.0032 for COP; *P* = 0.0178 for BER].

**Figure 2:**
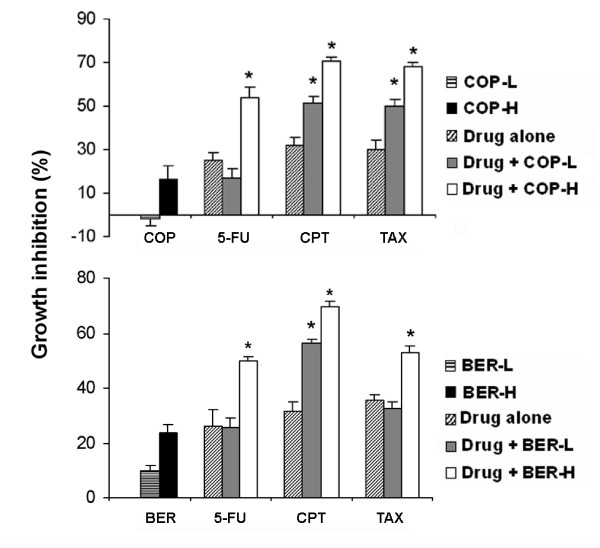
**Growth inhibition by combined use of COP/BER and chemotherapeutic agents in A549 cells.** Effects of COP or BER, combined with 5-fluorouracil (5-FU), camptothecin (CPT), or paclitaxel (TAX), on the growth of A549 cells. COP-L: 1.6 μg/mL; COP-H: 6.4 μg/mL; BER-L: 0.5 μg/mL; BER-H: 4 μg/mL. *P < 0.05, compared to drugs alone. Values are means ± SD of 4 independent assays.

Low and high doses of COP or BER combined with 5FU, CPT, or TAX were used to treat A549 cells to investigate the inhibitory effects of COP and BER in combination with chemotherapeutic drugs on cancer cells. As shown in Figure 2, a combination of a low dose of COP and chemotherapeutic drugs had an inhibitory effect stronger than CPT or TAX alone on cancer cell growth (*P* < 0.001 for both CPT and TAX), whereas high doses of COP enhanced the inhibitory effects of CPT, TAX, and 5FU on A549 cell growth (*P* < 0.001 for CPT, TAX, and 5FU). In addition, the combination of a high dose of BER with chemotherapeutic drugs exhibited an inhibitory effect stronger than CPT, TAX, or 5FU alone on A549 cell growth. These findings suggest a potential use of COP and BER as adjuvant therapies for NSCLC.

### Production of reactive oxygen species in A549 cells

**Figure 3:**
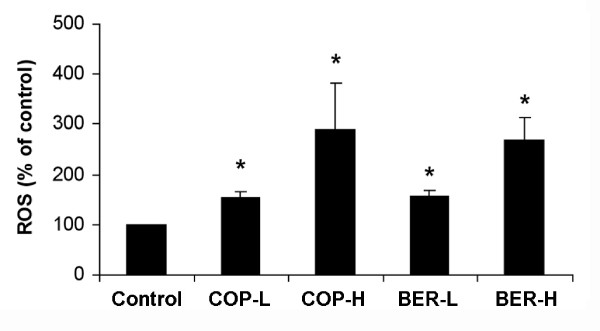
**Effects of COP and BER on ROS production in A549 cells. **Cells were treated with COP-L (1.6 μg/mL), COP-H (6.4 μg/mL), BER-L (0.5 μg/mL), or BER-H (4 μg/mL) for 24 h and harvested for ROS determination. * *P* < 0.05 compared to control. Values are means ± SD of 4 independent assays.

The intracellular levels of ROS production were measured after treatment with low or high doses of COP and BER. As shown in Figure 3, low doses of COP and BER increased ROS production by approximately 50% (*P* < 0.05), relative to the control group, and ROS production in the cells incubated with high doses of COP and BER was nearly 3 times that of the control group (*P* < 0.01, n = 4).

Our results show that both COP and BER significantly increased ROS levels in A549 cells, in a dose-dependent manner, and enhanced the inhibitory effect of chemotherapeutic drugs on A549 cells. The present study agrees with previous findings that ROS production is increased in cancer cells [21], to sensitize the cancer cells to drugs [13,22] and to radiotherapy [23]. It has been reported [24] that BER enhances the anticancer effect of irradiation by increasing ROS production in human hepatoma cells.

### Inhibition of MDR in A549 cells

**Figure 4:**
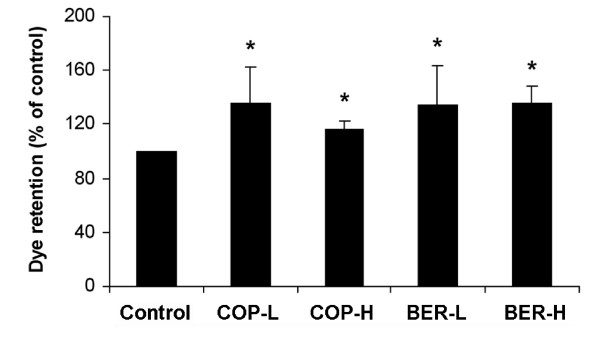
**Effects of COP and BER on MDR in A549 cells. **Cells were treated with COP-L (1.6 μg/mL), COP-H (6.4 μg/mL), BER-L (0.5 μg/mL), or BER-H (4 μg/mL) for 24 h and harvested for dye retention determination. * *P* < 0.05 compared to control. Values are means ± SD of 4 independent assays.

Rhodamine 123 retention in A549 cells was tested to determine whether COP and BER affect MDR. As shown in Figure 4, both low and high doses of COP and BER enhanced dye retention by as much as 40% (*P* < 0.05). Because elevated dye retention levels are inversely related to MDR [20], this suggests that the inhibitory effects of COP and BER on A549 cells were enhanced due to the prolonged intracellular retention of the chemotherapeutic drugs.

## Discussion

NSCLC is extremely difficult to treat because of its low therapeutic and long-term survival rates [11]. This study demonstrates that a combination of COP or its major constituent BER with chemotherapeutic drugs including 5-FU, CPT, and TAX exhibits a stronger inhibitory effect on the growth of A549 human lung cancer cells than any individual treatment. These findings suggest a potential use of COP and BER in the adjuvant treatment of NSCLC.

ROS levels are elevated in cells exposed to various stressors, including anticancer drugs, leading to apoptosis by stimulating pro-apoptotic signaling molecules (*e.g.*, P53, MAPK, *etc.*) [25]. Some studies [13,22] have shown that increasing the production of ROS may sensitize cancer cells to drugs. Our results show that both COP and BER significantly increase ROS levels in A549 cancer cells in a dose-dependent manner, which is consistent with the enhanced inhibitory effect of chemotherapeutic drugs combined with COP or BER on A549 cancer cells.

The anticancer effects of COP and BER may involve other pathways. Both 5-FU and CPT inhibit cellular DNA replication [26,27], and TAX inhibits cell division by stabilizing microtubules, triggering death of rapidly dividing cancerous cells [28]. Some studies [29,30] have indicated that COP and BER exhibit anticancer effects by inhibiting the activity of DNA topoisomerases and protein kinase C. Our previous study [9] showed that COP and BER markedly inhibit cell proliferation and induce apoptotic cell death of MCF-7 cells through up-regulation of interferon-β, an important cytokine that regulates cell growth and death. COP and BER also enhance the anticancer effect of estrogen receptor antagonists, including tamoxifen and fulvestrant, likely by regulating multiple cancer-related genes, *e.g.EGFRHER2bcl-2COX-2*, and *p21*[10]. In this study, we observed some differences in efficacy between COP and BER. It may be that there were components in COP other than BER that contributed to its anticancer effect in compliance with our previous studies [9,10]. Further studies are required to discover the pathways targeted by COP and BER.

## Conclusions

This study demonstrated that combinations of COP or BER with chemotherapeutic drugs (5-FU, CPT, and TAX) are more effective in inhibiting the growth of A549 cells than that of any single-agent therapy, possibly due to increased production of ROS and reduce MDR.

## Abbreviations

COP: Coptis extract; BER: Berberine; 5-FU: Fluorouracil; CPT: Camptothecin; TAX: Paclitaxel; NSCLC: Non–small cell lung cancer; ROS: Reactive oxygen species; MDR: Multidrug resistance; DMSO: Dimethyl sulfoxide; DCFH-DA: 2′,7′-dichlorofluorescein diacetate; DCF: Dichlorofluorescein; Pgp: P-glycoprotein.

## Competing interests

The authors declare that they have no competing interests.

## Authors’ contributions

JK conceived of the study, designed the study, and wrote the manuscript. CH designed the study, performed the experiments, analyzed the data and wrote the manuscript. RR performed the experiments and wrote the manuscript. JL performed the experiments, assisted the study design and data analysis. JW performed the experiments. KZ provided the materials and designed the study. All authors read and approved the final manuscript.
